# Development and validation of a physical activity questionnaire for patients following cardiac surgery

**DOI:** 10.3389/fresc.2026.1882995

**Published:** 2026-07-02

**Authors:** Benja Songsaengrit, Pajeemas Kittipanya-ngam, Montri Yasud, Panadda Yasud, Montana Donsom, Supattra Chantawong, Nichamon Ekphaphan, Worrawut Usupharach

**Affiliations:** 1 Operations Division, Queen Sirikit Heart Center of the Northeast, Faculty of Medicine, Khon Kaen University, Khon Kaen, Thailand; 2 Rehabilitation Medicine, Faculty of Medicine, Khon Kaen University, Khon Kaen, Thailand

**Keywords:** cardiac surgical procedures, cardiorespiratory fitness, motor activity, surveys and questionnaires, walk test

## Abstract

**Background:**

Physical activity questionnaires assess functional capacity in cardiovascular patients, yet none are specifically developed for second phase cardiac surgery rehabilitation. This study aimed to develop and validate a physical activity questionnaire for patients during the first three months following cardiac surgery.

**Methods:**

A cross-sectional study was conducted with 94 post-cardiac surgery patients within three months after the surgery. In Phase 1, a physical activity questionnaire was developed for these patients, and later, its content validity was examined, and its reliability was determined. In Phase 2 of the study, the validity of the questionnaire was tested by evaluating its correlation with the 6-minute walk distance (6MWD), the scores from the Duke Activity Status Index (DASI), and the dimension-specific scores of the SF-36 quality of life questionnaire.

**Results:**

A physical activity questionnaire for post-cardiac surgery patients was developed with 27 items. The Index of Item-ObjectiveCongruence (IOC) showed an average value of 0.91, and the reliability measured by Cronbach's alpha was 0.88. Regarding the validity, the scores from the questionnaire showed a significant correlation with the 6MWD (r^s^ = 0.58), the scores from the DASI (r^s^ = 0.69), and with the physical functioning dimension of the SF-36 questionnaire (r^s^ = 0.40).

**Conclusion:**

The physical activity questionnaire demonstrated the validity and reliability to assess the functional capacities of post-cardiac surgery patients, as well as correlation with other standardized physical activity questionnaires.

## 1 Introduction

Cardiovascular diseases are the leading cause of death in the world population (1), Cardiac rehabilitation is one of the important treatment for cardiovascular secondary prevention (2) because it helps improve exercise capacity. Exercise capacity can be assessed by exercise testing (3). Cardiopulmonary exercise testing is the gold standard (4), but the 6 min walk test (6MWT) is commonly used in post-cardiac surgery patients due to its practicality (5, 6). In prior research, 6-minute walk distance (6MWD) has a correlation with cardiopulmonary exercise testing in heart failure patients (7) and is associated with metabolic equivalent in patients within two months after coronary artery bypass grafting (8). Also, it is used to determine physical functioning to assess post-cardiac surgical recovery (9), to evaluate the effectiveness of cardiac rehabilitation (10), and to determine the exercise intensity prescription (11).

However, the physical activity tests are limited to use in some cases (12), the physical activity questionnaires, including New York Heart Association Classification (13), the Duke Activity Status Index (DASI) (14, 15), and the Specific Activity Scale (16, 17), are being widely used due to their convenience and low cost. The results from the physical activity questionnaires can be interpreted in various ways, such as maximal oxygen consumption (14, 18), metabolic equivalent (19), scores (20), and activity levels (13, 16). The 6MWD prediction from the physical activity questionnaires in post-cardiac surgery patients have not appeared in previous studies. Most studies predicted the 6MWD from the baseline demographic and the clinical characteristics (6, 21–23). More importantly, there has not been a physical activity questionnaire that has been specifically designed for patients in the second phase of cardiac rehabilitation. Therefore, this study aimed at developing and testing the validity and reliability of the physical activity questionnaire for post-cardiac surgery patients during their first three months after the surgery.

## 2 Methods

### 2.1 Study design

This cross-sectional study was conducted at Queen Sirikit Heart Center of the Northeast at Khon Kaen University's Faculty of Medicine in Khon Kaen Province, Thailand, from September 2022 to July 2024. This study has been reviewed and approved by the Khon Kaen University Ethics Committee for Human Research (HE641202), based on the Declaration of Helsinki and the International Council for Harmonisation Good Clinical Practice Guidelines.

### 2.2 Study participants

The participants were post-cardiac surgery patients attending follow-up outpatient treatment within 3 months from the date of discharge from the hospital. They were 18 years of age or older, were able to communicate verbally, were able to read and write well, and were willing to cooperate. Patients with contraindications or those having the inability to undergo the 6 min walk test 5 were excluded from the study. In addition, those with hearing problems, vision problems, or intellectual disabilities were excluded. In total, 94 patients volunteered to participate in this study. The sample size calculation was based on the correlation value of the 36-Item Short Form Health Survey (SF-36) questionnaire in the dimension of physical functioning and the 6MWD (r^s^ = 0.54) (9) with a power of test of 80% at a significance level of 5% (24).

### 2.3 Study protocols

**Figure 1:**
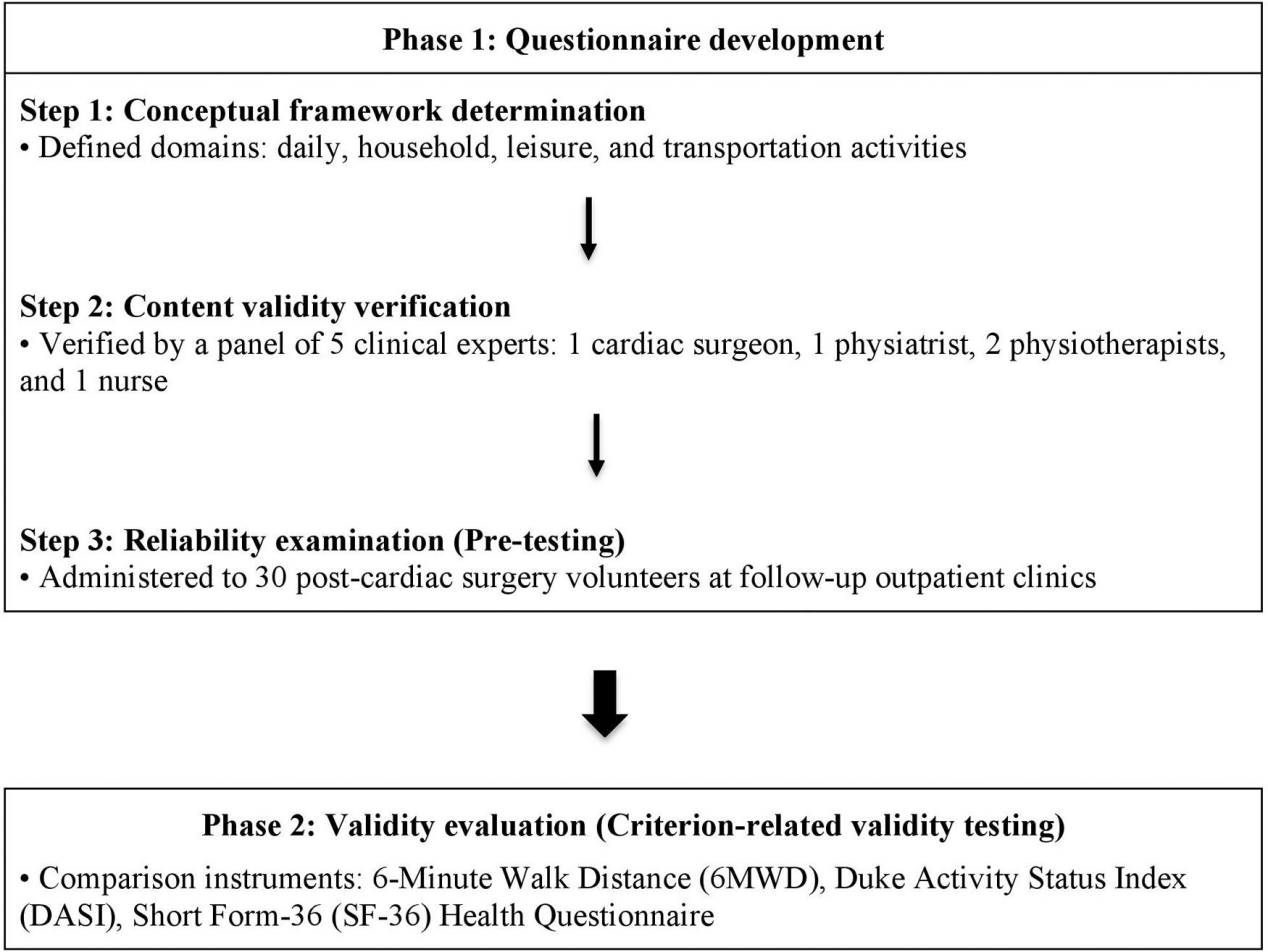
Flowchart of the study protocol.

The study was divided into two phases (Figure 1). In Phase 1, the physical activity questionnaire for post-cardiac surgery patients during their first three months of rehabilitation was created. After that, in Phase 2, the validity of the physical activity questionnaire that had been developed in Phase 1 was tested.

In Phase 1, there were 3 steps. Firstly, the conceptual framework of the patients' physical activities, including daily activities, household activities, leisure activities, and transportation activities, was determined in accordance with the activities related to the patients' health, culture, and well-being. Secondly, the content validity was verified by a group of 5 experts, who consisted of 1 cardiac surgeon, 1 physiatrist, 2 physiotherapists, and 1 nurse. Each of these experts had more than 5 years of experience in treating patients with cardiovascular diseases. Finally, the reliability was examined with 30 volunteers, who were post-cardiac surgery patients attending their follow-up outpatient treatments. These were the patients, who had undergone cardiac surgery and who fell within the 3-month period from the date of discharge from the hospital.

In Phase 2, the validity of the physical activity questionnaire was evaluated in accordance with its relationship to the 6MWD, the DASI, and the SF-36 questionnaire in each dimension.

### 2.4 Statistical analysis

All statistical analyses were conducted using SPSS Statistics 28.0 (25). The continuous data was presented as means and standard deviations, while the categorial data was presented in numbers and percentages. The process of evaluating the content validity was conducted using the index of item objective congruence method. The reliability was evaluated using Cronbach's Alpha coefficient. Regarding the validity testing process, the Shapiro–Wilk test was used to assess the normality of the data distribution. If the data followed a normal distribution, then the results were presented using the mean ± SD, and the relationships between the variables were tested using the Pearson correlation coefficient. However, If the data did not follow a normal distribution, then the results were presented using the median (IQR), and the relationships between the variables were tested using the Spearman rank correlation coefficient. The factors influencing the outcomes were analyzed using multivariate analysis, and the relationship equation was derived using linear regression analysis. The agreement analyses were performed, including the Intraclass Correlation Coefficient (ICC) using a two-way mixed-effects model with absolute agreement and a Bland-Altman analysis. Additionally, the significance level was set to 0.05.

## 3 Results

In Phase 1, a physical activity questionnaire specifically designed for patients in the 3-month post-cardiac surgery period was developed. The questionnaire consisted of 27 questions, 2 of which related to daily activities, 8 related to household activities, 5 related to leisure time activities, 7 related to transportation activities, and 5 related to occupational activities (Table 1).

**Table 1:** The physical activity questionnaire for patients in the 3-month post-cardiac surgery period.

Activities	Points
1.‏ Are you able to sit down to talk or eat?	
2.‏ Are you able to get dressed while sitting or standing?	
3.‏ Are you able to exercise your legs and arms in a sitting position?	
4.‏ Are you able to walk at a slow pace?	
5.‏ Are you able to walk at a normal pace?	
6.‏ Are you able to walk at a faster pace to go to work or class?	
7.‏ Are you able to walk while carrying an item weighing up to 10 kilogram?	
8.‏ Are you able to walk on uneven surfaces, such as rough paths, fields, or lawns?	
9.‏ Are you able to walk up the stairs?	
10.‏ Are you able to walk uphill?	
11.‏ Are you able to load clothes into the washing machine, hang-up clothes, or fold clothes?	
12.‏ Are you able to wash dishes?	
13.‏ Are you able to change the bed sheets?	
14.‏ Are you able to cook?	
15.‏ Are you able to sweep the floor?	
16.‏ Are you able to sweep the ground outside of the house?	
17.‏ Are you able to babysit?	
18.‏ Are you able to move items or furniture around?	
19.‏ Are you able to do agricultural work of a moderately intensity nature, such as feeding or walking animals?	
20.‏ Are you able to sweep fallen leaves?	
21.‏ Are you able to wash the car?	
22.‏ Are you able to loosen the soil?	
23.‏ Are you able to operate a grass trimmer or a walk-behind mower?	
24.‏ Are you able to drive a car or light truck?	
25.‏ Are you able to repair a car or motorcycle?	
26.‏ Are you able to work in an industrial facility with machinery?	
27.‏ Are you able to do construction work or home repairs?	
**Total score**	

Scoring criteria: N/A, not applicable; 0, unable to do; 1, able to do with assistance; 2, able to do independently.

The content validity testing of the questionnaire for patients during the 3-month post-cardiac surgery period, which was conducted by the 5 experts, showed that the average the Item-Objective Congruence value of all the questions had been 0.91 (between 0.8 and 1.0). Moreover, based on the experts' comments, the researcher further improved the questionnaire in order to make the questions clearer and easier to understand.

The reliability of the questionnaire was evaluated using the Cronbach's Alpha coefficient. The data, which was collected from 30 volunteers attending outpatient follow-up visits within three months after surgery, yielded a Cronbach's Alpha value of 0.88. This value indicated that the questionnaire was reliable and suitable for use in phase 2 (26).

In Phase 2, the validity testing of the questionnaire was conducted with 94 volunteers, who attended follow-up outpatient treatment within three months after their surgeries. These volunteers met the criteria for participation in the study and then completed the questionnaires by themselves. The demographic and clinical characteristics of the participants are presented (Table 2).

**Table 2:** Basic demographic and clinical characteristics of the participants.

Characteristics	Participants (*n* = 94), *n* (%)
Male sex	62 (66)
Age (years), median (IQR)	60 (52–67)
BMI* (kg.m^−2^), median (IQR)	22.2 (19.6–24.9)
Diagnosis	
Valvular heart disease	45 (48)
Coronary artery disease	33 (35)
Congenital heart disease	10 (11)
Valvular heart disease with coronary artery disease	6 (6)
Comorbidity	
No comorbidity	18 (19)
Diabetes mellitus	28 (29)
Hypertension	67 (71)
Dyslipidemia	31 (33)
Chronic kidney disease	2 (2)
Arterial fibrillation	18 (19)
Pulmonary	3 (3)
Rheumatologic	3 (3)
Other	9 (9)
Ejection fraction (%), median (IQR)	58 (46–67)
Type of surgery	
CABG*	39 (41)
Non-CABG	55 (59)
Complications	
No complication	25 (26)
Cardiovascular	63 (67)
Pulmonary	10 (10)
Infection	0 (0)
Other	4 (4)

BMI, body mass index; CABG, coronary artery bypass graft.

The scores that the participants had received from the questionnaire were correlated with the 6MWD, the scores from the DASI, and the scores from the SF-36 questionnaire, specifically with regard to the dimensions of physical functioning, general health, and reported health transition (Table 3).

**Table 3:** Correlations between the physical activity questionnaire score for patients in the 3-month post-cardiac surgery period and other outcome measures (*n* = 94).

Outcome measures	r^s^ (95% CI)	*P* value
The 6-Minute Walk Distance	0.58 (0.42, 0.70)	<.001
Duke Activity Status Index	0.69 (0.57, 0.79)	<.001
36-Item Short Form Health		
Survey subscales		
Physical functioning	0.40 (0.21, 0.56)	<.001
Role physical	0.16 (−0.04, 0.36)	.107
Bodily pain	0.12 (−0.09, 0.32)	.248
General health	0.25 (0.04, 0.43)	.015
Vitality	0.04 (−0.16, 0.25)	.654
Social functioning	−0.01 (−0.22, 0.19)	.883
Role-emotional	0.04 (−0.16, 0.25)	.658
Mental health	−0.04 (−0.24, 0.16)	.695
Reported health transition	0.24 (0.03, 0.42)	.019

r^s^, Spearman correlation coefficient.

Along with sex, age, and diagnosis, the scores from the questionnaire were factors that had influenced the 6MWD with an adjusted R square value of 0.49 (Table 4). The following equation was derived:

The6MWD=218.14+Male(40.64)+Age(−1.72)+Valvularheartdisease(51.02)+Physicalactivityquestionnairescores(3.22)

**Table 4:** Multivariate analysis for the physical activity questionnaire score for patients in the 3-month post-cardiac surgery period associated with each factor.

	Unstandardized Coefficients	Unstandardized Coefficients		*P*-value	Part correlation
	B	Standard error	Beta		
(Constant)	218.140	36.730	NA	<.001	NA
Male	40.636	13.146	0.242	.003	0.228
Age, years	−1.723	0.455	−0.297	<.001	0.279
Valvular heart disease	51.021	12.265	0.319	<.001	0.307
Physical activity questionnaire score	3.220	0.570	0.454	<.001	0.417

Notes: Age is in years, Sex is coded as Male = 1 and Female = 0, and Diagnosis is valvular heart disease.

**Figure 2:**
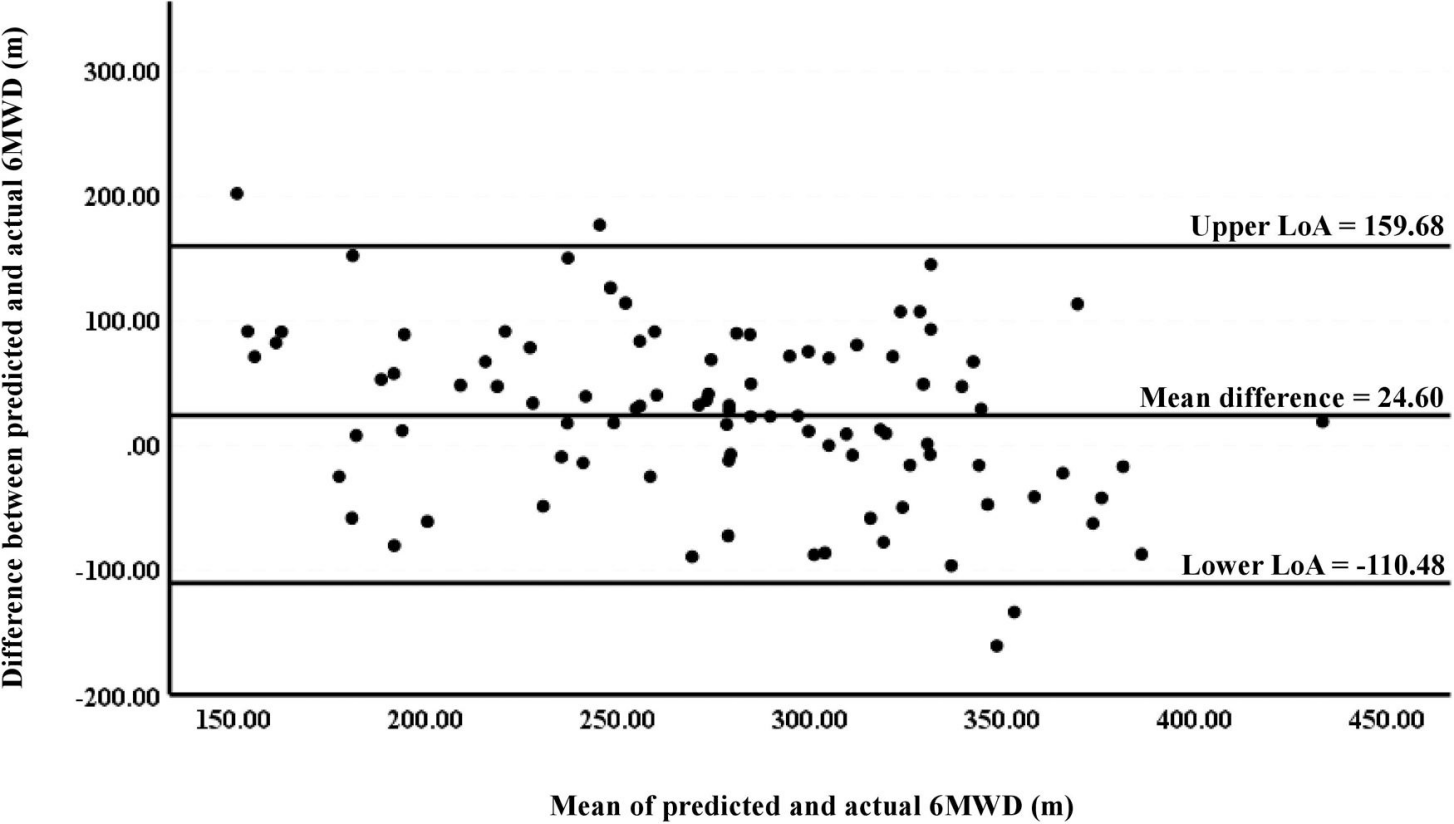
Bland-Altman plot comparing the questionnaire-predicted 6MWD and the actual 6MWD.

The single-measure ICC between the questionnaire-predicted 6MWD and the actual 6MWD was 0.49 (95% CI: 0.31–0.64, *p* < 0.001). Furthermore, the Bland-Altman plot showed a mean difference of 24.60 meters (95% LoA: −110.48 to 159.68), with over 95% of points within the limits, confirming acceptable consistency with the reference standard (Figure 2).

## 4 Discussion

This study developed a physical activity questionnaire specifically for patients in the 3-month period following cardiac surgery. The questionnaire was correlated with a submaximal test. Furthermore, an equation to predict the 6MWD based on sex, age, diagnosis, and physical activity questionnaire scores was derived.

The questionnaire demonstrated good reliability. It is a simple, easy-to-complete, self-administered tool for post-cardiac surgery patients who are unable to undergo exercise testing. It can also be used in settings with limited space. The questions assess the ability to perform general daily activities that are appropriate and culturally relevant to this patient group. The questionnaire was developed with a three-level scoring system for each item, which may serve as a crucial factor in the design of future research questionnaires (27).

There are many physical activity questionnaires for cardiac patients. The New York Heart Association classification questionnaire assesses physical activity for patients with heart failure. The assessment relies on the physician's judgment which can lead to variability between physicians (28, 29). The Specific Activity Scale questionnaire classifies into four levels based on metabolic equivalent, which is like the New York Heart Association classification. Its reproducibility with the New York Heart Association classification questionnaire was 56% (16). The DASI questionnaire provides a practical and structured way to assess physical activity capacity through common daily activities. The results are summarized into a DASI score (14). The Veterans Specific Activity Questionnaire is similar in structure but focuses primarily on exercise-related activities. It assesses whether activities can be performed without or with minimal symptoms, with results presented as the maximum metabolic equivalent achieved (19). Similarly, the Specific Activity Questionnaire evaluates whether the activity can be performed without symptoms (18). However, post-cardiac surgery patients prioritize physical activities that are related to work over sports and sexual activity (30).

This study discovered a correlation between the physical activity questionnaire score and the 6MWD (r^s^ = 0.58), which was consistent with previous studies. In those studies, among patients also in the 3-month post-cardiac surgery period, the scores from the SF-36 questionnaire in the physical functioning dimension were found to correlate with the 6MWD (r^s^ = 0.54) (9). Some of the questions in the physical activity questionnaire for patients in the 3-month post-cardiac surgery period were similar to those in the SF-36 questionnaire in the physical functioning dimension within a similar time frame. Furthermore, this study found a correlation between the physical activity questionnaire and the DASI questionnaire scores (r^s^ = 0.69), as well as the SF-36 questionnaire in the physical functioning dimension (r^s^ = 0.40) and the reported health transition dimension (r^s^ = 0.24). Consistent with previous studies, a correlation was found between the DASI questionnaire score and the SF-36 questionnaire score in the physical functioning dimension (*r* = 0.79) in those patients with a previous myocardial infarction (31). Moreover, the SF-36 questionnaire score in the physical functioning dimension and the DASI questionnaire score were found to correlate with metabolic equivalent (32), exercise capacity, and maximal oxygen consumption in patients with cardiovascular disease (33).

This study revealed that sex, age, diagnosis, and the physical activity questionnaire scores for patients during the 3-month post-cardiac surgery period can predict the 6MWD during the first 3 months after hospital discharge following cardiac surgery. The findings from this study confirmed the results from previous research studies given that factors, such as sex and age, can predict the 6MWD in post-cardiac surgery patients (6, 21–23). Also, this study found that the diagnosis and the physical activity questionnaire score for patients during the 3-month post-cardiac surgery period can predict 6MWD, which, in turn, can predict the risk of mortality (34) and can assess changes in functional capacity after cardiac rehabilitation (35) in post-cardiac surgery patients. Additionally, the supplementary agreement analyses confirmed acceptable clinical consistency between the questionnaire-derived estimates and the actual 6MWD. This evidence further strengthens the overall validity and utility of the developed instrument for post-cardiac surgery patients.

Three limitations were identified in this study. Firstly, although the American Thoracic Society Statement recommends a 30-meter track for the 6MWT, a 15-meter track was used. However, previous studies showed that the 6MWT on both 15-meter and 30-meter tracks in patients with chronic lung diseases had yielded similar walking distances within 6 min (36). Secondly, this study did not utilize the cardiopulmonary exercise testing method, which is a gold standard for assessing exercise capacity. However, cardiopulmonary exercise testing requires a variety of equipment, and a trained operators (37). Thirdly, the physical activity questionnaire in this study was only able to assess physical activity capacity during the first 3 months after surgery and was unable to evaluate physical activity capacity in the long term. Future research should address these initial limitations so that the physical activity questionnaire for post-cardiac surgery patients can be further developed.

The physical activity questionnaire can be used to screen for physical activity in early postoperative patients, particularly in settings where formal exercise testing is not yet available. Medical personnel can use this questionnaire to stratify patients' risk and tailor the intensity of their rehabilitation program. It can also support ongoing progress monitoring, allowing for timely adjustments to treatment goals and interventions. Furthermore, this tool can be easily integrated into a multidisciplinary cardiac rehabilitation program, enabling physical therapists, nurses, and physicians to work together to optimize recovery.

## 5 Conclusion

This study demonstrated that for post-cardiac surgery patients, the newly developed self-administered physical activity questionnaire had been deemed valid without the need for an exercise test. Furthermore, it was determined to be reliable and consistent with other standardized questionnaires that are currently being used to assess the capacity for physical activity in patients within the first 3 months after cardiac surgery.

## Data Availability

The raw data supporting the conclusions of this article will be made available by the authors, without undue reservation.
